# Grand Challenges in Genome Editing in Plants

**DOI:** 10.3389/fgeed.2020.00002

**Published:** 2020-04-24

**Authors:** Bing Yang

**Affiliations:** ^1^Division of Plant Sciences, Christopher S. Bond Life Sciences Center, University of Missouri, Columbia, MO, United States; ^2^Donald Danforth Plant Science Center, St. Louis, MO, United States

**Keywords:** ZFN, TALEN, CRiSPR/Cas, agriculture, challenge, plants, genome editing

The world depends on agriculture, and agriculture itself faces grand challenges, such as meeting the needs for food, fiber, and bioenergy required by the continuously increasing human population. It is necessary to meet these needs using the shrinking arable land area available whilst also adapting to the climate changes that lead to more frequent flooding, drought, and high temperatures, as well as breeding crop plants that are able to withstand diseases and pests with little use of environmentally harmful chemicals. All these challenges call for innovative solutions that will be derived from fundamental and applied research, including genome editing, in plant sciences, and agriculture.

Genome editing refers to enabling precise genome modifications, such as site-specific insertion, deletion, substitution, and epiallelic changes of the DNA in a cell or organism. Genome editing is basically based on the *in vivo* DNA double strand breaks (DSBs) induced by engineered endonucleases that are programmed to recognize the preselected genomic sites and exploitation of cellular DSB repair mechanisms (Carroll, 2014). The programmable nucleases include meganucleases (Gong and Golic, 2003), zinc finger nucleases (ZFNs) (Urnov et al., 2005), transcription activator-like effector nucleases (TALENs) (Christian et al., 2010; Li et al., 2011), and clustered regularly interspaced short palindromic repeats (CRISPR) associated nucleases (Cas) (Jinek et al., 2012; Cong et al., 2013; Mali et al., 2013). Additionally, engineered nuclease variants can perform genome editing without DSBs (e.g., through causing DNA single strand breakage) (Rees and Liu, 2018) or epigenome editing (no DNA breakage at all and no DSB repair) (Holtzman and Gersbach, 2018).

Genome editing has become the most important biotechnological tool that has spurred the growth of our biological knowledge and the field of biotechnology itself with tremendous contributions that have enabled rapid progress in industry, medicine, and agriculture. In the past 10 years, we have witnessed the rapid advance in the development of CRISPR-based genome editing technologies and their applications in a variety of fields, including plant functional genomics and crop improvement. The genome editing technologies in plants include engineering of sequence-specific nucleases, delivery of editing reagents, generation and selection of editing events, and characterization and utilization of intact plants, which have further implications for public acceptance and regulatory approval of the genome edited plants. Over the last decade, genome editing platforms have been established and applied in more than 45 genera of plants (Shan et al., 2020). However, the need for better understanding of the molecular and genetic mechanism of genome editing, continuing improvements, and novel applications of genome editing technologies in plants still require major research endeavors. Specifically, plant genome editing faces major challenges as outlined below.

## Overcoming Species- and Genotype-Dependent Transformation

Unlike in animals, genome editing in plants requires plant transformation that involves delivering of the editing reagents into plant cells, selection of edited cells, and regeneration of intact plants with desired edits. For most crops, transformation and regeneration still remain strenuous despite more than three decades of technological advances. They are therefore a bottleneck in fully materializing the great potential of genome editing in agriculture (Altpeter et al., 2016). Recently, studies have used the development regulator (DR) genes (e.g., *Baby Boom* and *Wuschel2*) along with optimal regimes of phytohormones to improve transformation efficiency in plants. Expression of specific combinations of the DR genes in plant somatic cells leads to the formation of meristematic tissue capable of regenerating shoots (Lowe et al., 2016; Maher et al., 2020). However, further improvement and widespread adaption of the technology for crop plants beyond the few tested remain challenging. Research endeavors also need to make new technological advances amenable to the various forms of genome editing, including targeted mutagenesis, gene insertions, and base replacements.

## Broadening Targets for Crop Improvement Through Genome Editing

The great promise of genome editing in agriculture can only be manifested through fine-tuning the gene or genetic element of interest in crop plants (Kwon et al., 2019; Oliva et al., 2019). However, the number of targets for crop improvement through genome editing are currently limited due to a lack of understanding of biological process involving genes, pathways, networks, and their interactions with environmental factors. On the other hand, CRISPR-based genome editing technologies start to show the feasibility of performing genome-wide and high throughput functional genomics, expediting gene/trait discovery in model or non-model crops (Lu et al., 2017; Meng et al., 2017). The challenge is to integrate multidisciplinary approaches to identify targets that both bear potential for beneficial agronomic traits and are suitable for genome editing (Araus et al., 2018).

## Achieving High-Efficiency Base Replacement

The majority of genome editing development and application focuses on targeted mutagenesis. In contrast, the methods and applications for the base replacement remain very limited, particularly in plant genome editing due to the very low efficiency in some plants and lack thereof in the majority of crop plants. In crop plants, superior traits are often governed by genetic variations in SNPs (single nucleotide polymorphisms); the challenge is to identify them and integrate them in elite varieties in an expedited manner. This provides a great opportunity to recapitulate the superior alleles in the target genomes through base replacement. The HDR (homology directed repair) based base replacement or gene insertion holds promise in this. The recently reported “prime editing” technology that utilizes Cas9 and guides RNA variants to precisely alter a specific DNA sequence also holds promise in base replacement if adapted and validated in plants (Anzalone et al., 2019). Prime editing is superior in some instances over the base editors which convert C to T and A to G within a window of a guide RNA specified DNA site because it offers the possibility of replacement of a small genomic region (Rees and Liu, 2018). Research endeavors are needed to adapt and improve these technologies for a wide range of plant species.

## Toward Better Public Perception and Regulatory Oversight of Genome Editing in Agriculture

Genome editing technologies can be used to create genotypic and phenotypic variations in plants that are indistinguishable from those obtained through natural means or conventional mutagenesis approaches; therefore, the genome edited products do not readily fit current definitions of genetically modified organisms (GMOs) within most regulatory regimes (Wolt et al., 2016). The usage of genome editing can help breed crops within a shorter time and with more precision, maximize the genetic potential of crop yields, develop germplasm that is more tolerant to pests, diseases, and other abiotic stresses, and help prolong shelf life of food products to avoid food waste. Therefore, the challenge would be to find greater public and regulatory acceptance of genome editing over the use of transgenic approaches. It is crucial to strengthen public trust and influence regulatory frameworks, which in turn will impact the utility of genome editing in agriculture, and which entails promoting transparency—sharing and openness of information regarding genome editing techniques and applications.

The goal of *Genome Editing in Plants* is to publish all findings and experiences in plant genome editing and epigenome editing, including experimental, analytical, and theoretical methodologies and resources in plant genome editing, as well as studies related to the biological function of genes and networks in plants using genome editing technologies. The journal also aims to publish high-impact, peer-reviewed commentary articles and review articles in the area of genome editing in plants.

## Author Contributions

The author confirms being the sole contributor of this work and has approved it for publication.

## Conflict of Interest

The author declares that the research was conducted in the absence of any commercial or financial relationships that could be construed as a potential conflict of interest.
